# A-Situ: a computational framework for affective labeling from psychological behaviors in real-life situations

**DOI:** 10.1038/s41598-020-72829-3

**Published:** 2020-09-28

**Authors:** Byung Hyung Kim, Sungho Jo, Sunghee Choi

**Affiliations:** School of Computing, KAIST, Daejeon, Republic of Korea

**Keywords:** Health care, Computer science, Emotion

## Abstract

This paper presents a computational framework for providing affective labels to real-life situations, called A-Situ. We first define an affective situation, as a specific arrangement of affective entities relevant to emotion elicitation in a situation. Then, the affective situation is represented as a set of labels in the valence-arousal emotion space. Based on psychological behaviors in response to a situation, the proposed framework quantifies the expected emotion evoked by the interaction with a stimulus event. The accumulated result in a spatiotemporal situation is represented as a polynomial curve called the affective curve, which bridges the semantic gap between cognitive and affective perception in real-world situations. We show the efficacy of the curve for reliable emotion labeling in real-world experiments, respectively concerning (1) a comparison between the results from our system and existing explicit assessments for measuring emotion, (2) physiological distinctiveness in emotional states, and (3) physiological characteristics correlated to continuous labels. The efficiency of affective curves to discriminate emotional states is evaluated through subject-dependent classification performance using bicoherence features to represent discrete affective states in the valence-arousal space. Furthermore, electroencephalography-based statistical analysis revealed the physiological correlates of the affective curves.

## Introduction

Emotion with supervised training datasets has received much attention in recent years, because it facilitates the understanding of emotional interactions between humans and computers by measuring emotional states such as joy, excitement, and fear. However, obtaining a massive amount of well-labeled data is usually very expensive and time-consuming. Although there have been advances in the annotation of emotional states in various environments, most cases depend on the participant’s self-assessment^1–3^. Apart from some existing issues with validity and corroboration^4^, this kind of reporting can only gather immediate human affective output in numerical form, providing only a limited understanding of complex emotional conditions and affective dynamics in daily life. Hence, it is critical to provide an automatic method for labeling human emotions elicited in real-life situations.

However, quantifying emotional responses based on the understanding of emotional interactions in real-world situations is challenging. It requires a cognitive understanding of the real-world objects that humans interact with and a determination of the expected affective level of the humans emotions based on the interaction. In response to this challenge, we start by defining the term “affective situation,” as a specific arrangement of affective entities in a spatiotemporal domain. Affective entities can be any of the real-world objects that people encounter and interact with in a place at a given time. Next, we present a computational framework to model and represent affective situations for labeling of real-life situations, called A-Situ. To model affective situations, the system derives pairs of emotion labels in the valence-arousal space from low-level features extracted from a psychological behavior sequence in a target situation.

Our model is mainly intended to estimate emotional adaptability to a situation in order to label emotional states underlying (1) affective response, (2) approach and withdrawal motivation, and (3) self-contentment, based on the extracted features in a sequence. While several methods^5^ have been proposed to record a wide range of emotions, participants affective responses have been mapped onto the valence and arousal coordinate system that has a parabolic-like shape resembling the 2-D emotion space^6,7^. Inspired by this phenomenon, the proposed framework represents an affective situation as a polynomial curve called the “affective curve,” which is fitted to a set of points over the valence-arousal emotion space. Furthermore, we aim to model and represent affective situations in real-world environments. To gather such environmental information, we design a wearable device that can be comfortably worn to allow users to act freely in everyday situations. consisting of a frontal camera, an accelerometer, and small physiological sensors. We use the data collected from our device to learn and represent affective situations and to provide proper affective labels to support learning of physiological changes in emotion recognition. Furthermore, modeling affective situations allows us to understand life content or material in human interaction, and representing these situations can determine the level of a persons expected feeling based on the interaction.

The distinct contributions of A-Situ, in contrast with existing systems are as follows:

- *Affective situation representation*: We introduce a polynomial curve called the “affective curve,” which is a set of cumulative points on the valence-arousal emotional space over time in a situation and represents affective dynamics in real-world environments.
- *Affective situation modeling*: Given a psychological behavior sequence in a given situation, we detect the expected feeling and track its changes. To model changes in the situation, we present three components: motivation, motion, and contentment. They reflect emotional responses to a situation’s underlying low-level features.
- *Physiological experiments to validate the effects of affective labels produced by A-Situ as ground truth*: We evaluate the proposed system over a long time series of life-logging data, affective situation dataset, covering multiple days in real-world scenarios. The evaluation involves investigating and analyzing the characteristics of brain signals related to different affective labels. Electroencephalography (EEG) based statistical analysis reveals that physiological responses correlate to continuous affective labels.

## Background

### Emotion

Multiple studies have been proposed to understand emotion and identify the different types of emotions people experience. During the last century, the two most widely accepted theories in affective intelligence are basic emotion and dimensional theories. Ekman and Plutchik proposed that core emotions have evolved through natural selection from categorical perspectives^8^. Ekman identified six basic emotional expressions: happiness, sadness, disgust, fear, surprise, and anger. Recently, social functions became a threshold to distinguish between anger and disgust, and between fear and surprise. Plutchik proposed a wheel of emotions, which illustrates eight basic emotions: joy, trust, fear, surprise, sadness, anticipation, anger, and disgust.

According to Bradley^9^ and Russel and Mehrabian^10^, human emotion can be conceptualized in three major dimensions of connotative meaning: valence (V), arousal (A), and dominance (D). Valence refers to the type of emotion and characterizes emotional states or responses ranging from unpleasant or negative feelings to pleasant, happy, or positive feelings. Arousal is the intensity of emotion and characterizes emotional states or responses ranging from sleepiness or boredom to frantic excitement. Dominance distinguishes emotional states having similar valence and arousal, ranging from “no control” to “full control”. For instance, the emotions of grief and rage have similar valance and arousal values but different dominance values. The entire scope of human emotions can be represented as a set of points in the three-dimensional (3D) VAC coordinate space.

**Figure 1:**
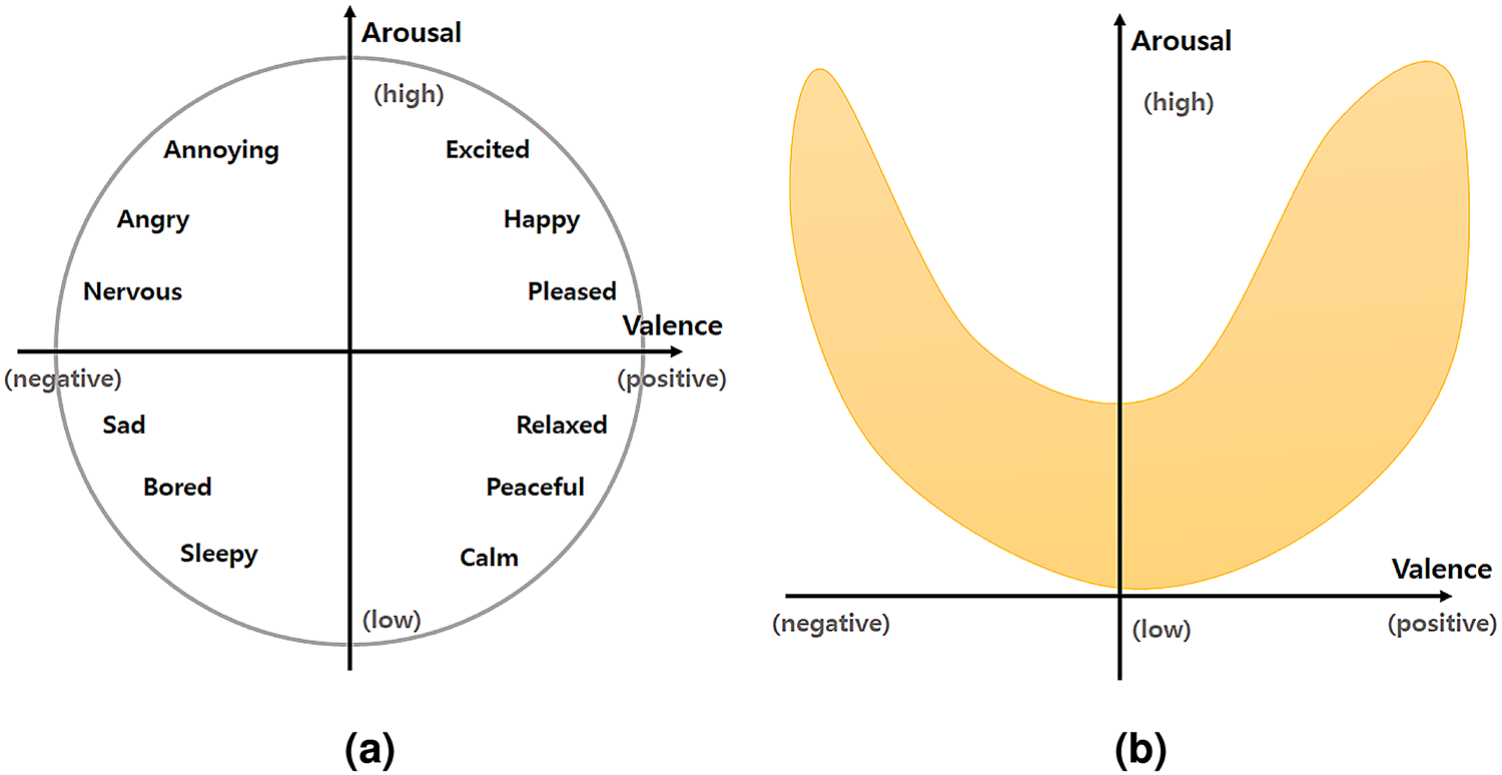
(**a**) Distribution of emotions in valence-arousal (V-A) space. (**b**) Parabolic shape of the V-A emotion space.

Conversely, each basic emotion can be represented as a bipolar entity^11^, characterizing all emotions by valence and arousal, and different emotional labels can be plotted at various positions on this two-dimensional VA plane. Label points on the two dimensions has been used to distinguish emotions such as sad and happy, representing a single emotion (see Fig. 1). Although several studies aim to collect a wide range of emotions using audio-visual content^12,13^, recent studies have found that affective responses mapped onto the emotional coordinate system are roughly parabolic (see Fig. 1b)^14,15^. For example, Dietz and Lang^16^ used the parabolic surface to assign temperament, mood, and emotion to define the personality of an affective agent.

### Affect labeling: explicit and implicit methods

Providing labels with emotional tagging enhances multi-disciplinary research areas, bridging the semantic gap between the low-level multimodal inputs such as images, videos, and texts and the high-level context-sensitive interpretation of emotion. For instance, providing reliable affective labels on a person’s behaviors measured by images and EEG signals helps to understand the behaviors in physiological and psychological perspectives. It quantifies affective responses to stimuli underlying affect dimensions in two ways: explicit and implicit tagging.

The explicit approach requires explicit actions to users to report their feeling in response to given events or stimuli. For instance, the international affective picture system (IAPS) has been a popular dataset for explicit tagging^5^. Participants manually measure their emotional response to a wide variety of stimuli associated with valence from positive to negative, arousal from high to low, and dominance from low to high using a self-reporting tool such as the self-assessment manikins (SAM). Dynamic assessments, such as ambulatory assessment^1^ and ecological momentary assessment^3^, allow the opportunity to assess contextual information about a behavior, and serve as real-time self-report methods to measure behavior and experiences in peoples daily lives. Affective labels obtained from explicit self-reporting tools have been considered ground-truth data for emotional states^7^ and used to build reliable emotion recognition systems^17^. At the same time, a major drawback of the explicit approach to labeling human emotions is the intrusiveness of the reporting procedure. Furthermore, obtaining a massive amount of hand-labeled data is very expensive and time-consuming.

Conversely, the implicit affective labeling approach is unobtrusive, as labeling is obtained by exposing users to stimuli and recording their responses. Therefore, implicit tagging does not require users to tag their emotional states and thus is a promising solution to overcome the limitation of the explicit tagging approach. In emotion recognition work, visual and motion features have been important elements for tagging emotions in different types of multimedia data, such as images and videos. Simmons et al.^18^ studied object motion as a visual feature in response to human affect and showed that increasing the motion intensity could also lead to increased levels of emotional arousal. Zhang et al.^19^ developed a method to characterize arousal using motion intensity and shot change rate in video clips. Hanjalic et al.^6^ used motion activity to determine arousal levels and represented continuous change of arousal as a curve. However, implicit methods like these have limits as far as a cognitive understanding of the real-world objects that humans interact with, since they have perceived emotions based on the scene as “understanding”.

### Psychological behaviors

An alternative to the above implicit approaches is to extract emotional features of psychological behaviors and associate them with emotional states. In this paper, we focus on developing psychological components in response to stimuli. Approach-avoidance theory describes action tendencies in response to emotion evoked by a stimulus event. The main proposition of the theory is that approach tendencies emerge toward positive stimuli and avoidance tendencies for negative stimuli. Krieglmeyer and Deutsch^20^ conducted experiments to compare measures of approach-avoidance behaviors in terms of the sensitivity and criterion-validity: moving a manikin on the screen towards and away from stimuli (manikin task), pulling and pushing a joystick (joystick task). From the improved latencies of correct responses of compatible and incompatible trials, they found the sensitivity and criterion-validity of the measures depends on the operationalization of the emotional behaviors. Their experimental results showed that a manikin task outperformed joystick tasks in this regard due to the means of distance change, such as (the manikin) running towards the object instead of (the joystick) moving it.

Many studies have proposed methods to label emotional difference based on psychological behaviors. For example, arm movements such as flexion and extension have been investigated to reveal positive and negative interactions between emotional stimuli and responses to approach and avoidance behaviors^21^. Seibt et al.^22^ used a joystick to determine whether positive and negative stimuli facilitate approach and withdrawal behaviors, respectively. Participants were instructed to control the joystick by either pulling it to increase the size of the stimuli or pushing it to decrease the size. However, the studies cited here are restricted to controlled experimental settings, require the use of specific equipment, and use limited-perception tasks in which participants are not interacting in real time with the system. In contrast, our system aims to label emotions by detecting the expected feeling and tracking its changes from a psychological behavior sequence in real-world situations.

### Physiological sensors in emotion recognition

Physiological measurement has been a key to understanding emotions. EEG measurement refers to the recording of the brain’s electrical activity with multiple electrodes placed on the scalp. Its very high temporal resolution is valuable to real-world applications despite its low spatial resolution on the scalp^23^. Moreover, mobility techniques of non-invasive EEG have extended their usage to the field of brain–computer interfaces (BCIs), external devices that communicate with the users brain^24^. Peripheral physiological signals such as skin conductance, heart rate, and breathing rate have been also carried out in emotion assessment^25^. In these measurements, distinct or peaked changes of physiological signals in the autonomic nervous system (ANS) elicited by specific emotional states at a single instantaneous time have been considered as candidates. Related studies on classifying emotional valence and arousal advanced significantly in many ways over the past few decades^26^. For instance, EEG signal has been widely used to develop wearable biosensors due to its simplicity in daily life applications^27^. However, this approach is limited and cannot be used to fully describe emotion elicitation mechanisms because of their complex nature and multidimensional phenomena. In our work, EEG is the most suitable choice among available physiological measurements since it measures the brain dynamics that control thoughts, feelings, and behaviors.

## Real-world data collection

**Figure 2:**
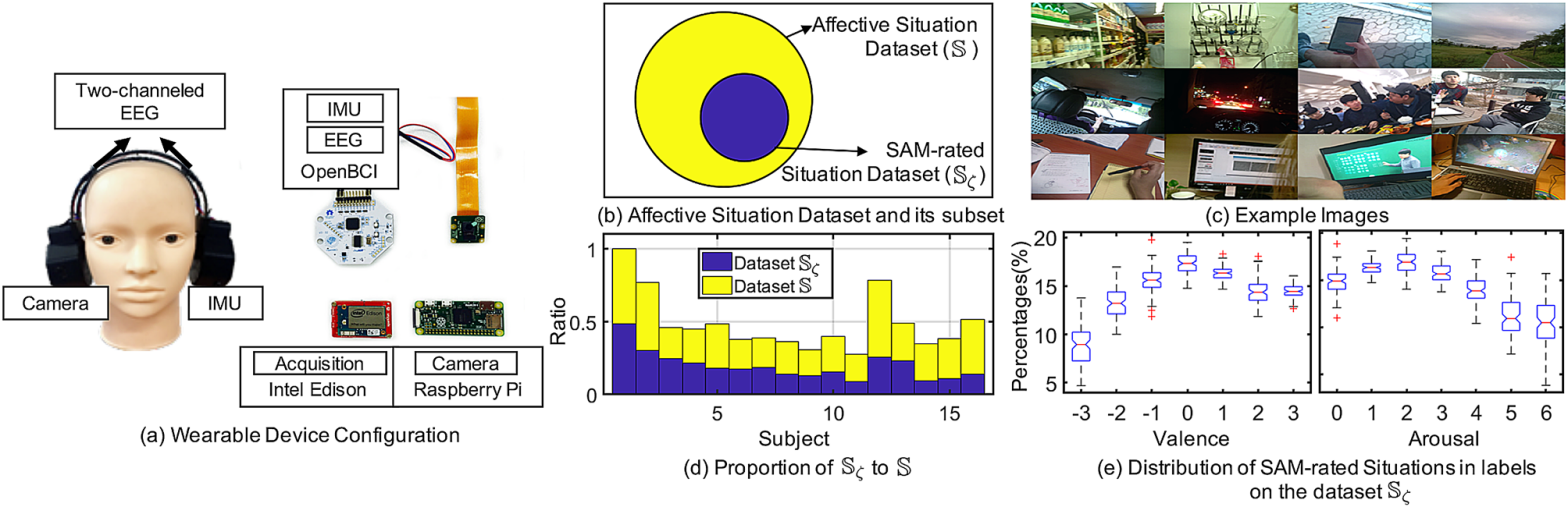
Wearable device configuration and overview of the Affective Situation Dataset. (**a**) Wearable device configuration. The location of two electrodes (F3, F4) on the 10–20 international system. (**b**) Affective situation dataset $\mathbb {S}$ and its subset $\mathbb {S}_{\zeta }$ which contains SAM-rated situations. (**c**) Example images from the dataset $\mathbb {S}$. (**d**) Proportion of the subset $\mathbb {S}_{\zeta }$ and the dataset $\mathbb {S}$. $N_s = 378$ for the subject 1. (**e**) Distribution of SAM-rated situations in valence and arousal labels on the subset $\mathbb {S}_{\zeta }$.

To evaluate the performance of our system for labeling emotion, we conducted real-world experiments on university life. An wearable device was designed (detailed information is described in Supplementary Materials) and distributed to participants to gather frontal images, EEG signals, and accelerometer signals in their daily life (Fig. 2a). The participants were 13 male and three female students aged 22–35 (27.3 ± 4.53) years. They evaluated our system in a real-world experiment related to school life (Fig. 2c) and performed more than one common task of a university student, such as taking/teaching classes, conducting research, or having discussions with colleagues. The participants were required to wear our device for 6 h per day in their daily work environment, for up to 45 days, with $10 compensation per day. They were asked to engage in free, normal activity over the course of their days. While wearing the device, affective situations are constructed and labeled as pairs of valence and arousal ratings on an affective curve. To evaluate the performance of our system, the participants performed self-assessment of their valence and arousal levels in relation to the affective situations using the web-based SAM, scaled from 0 to 6 for arousal and − 3 to 3 for valence (Fig. 2e). This procedure was approved by the KAIST Institutional Review Board (IRB) in Human Subjects Research. All research was performed in accordance with the relevant guidelines and regulations. Informed consent was obtained from all participants.

### Affective situation dataset

The affective situation dataset $\mathbb {S} = (\mathscr {S}^1, \ldots , \mathscr {S}^{N_s})$ is a set of affective situations collected using the above procedure. Subset $\mathbb {S}_{\zeta } \subset \mathbb {S}$ (Fig. 2b), which has a pair of valence ($\mathscr {V}$) and arousal ($\mathscr {A}$) ratings rated by the SAM, consists of the SAM-rated situation dataset (Fig. 2d). The affective labels ($\mathscr {V},\mathscr {A}$) of situations were used as ground truth to estimate the parameters of our system and evaluate its performance. The duration *T* of all situations was determined manually by three annotators, who spent 2.4 (± 1.2) min per situation. The inter-rater reliability was measured using interclass correlation (ICC); the result was 0.78. The average duration from the three annotators was ultimately used for each situation. Table 1 is detailed information on the affective situation dataset.

**Table 1:** Overview of the dataset contents.

Number of participants				16
Avg. number of durations (min) per situation				17.4
Rating values				Valence: − 3 to 3
				Arousal: 0 to 6
Recorded signals				2-channel EEG
				Frontal images
				Accelerometer

User #	Number of			
	Days	Emotional contents	Situations ($\mathbb {S}$)	Rated situations ($\mathbb {S}_{\zeta }$)
1	45	44	378	188
2	33	27	302	135
3	21	31	180	84
4	14	17	176	81
5	17	22	187	87
6	33	20	155	68
7	18	15	161	74
8	24	35	158	64
9	19	33	145	58
10	17	27	157	55
11	13	19	142	41
12	38	37	297	94
13	27	22	189	91
14	21	24	155	34
15	18	27	161	28
16	15	21	188	24

## Results

We evaluated the performance of our system for labeling emotions compared with the labels rated by the SAM. Furthermore, the distinctiveness of EEG signals categorized by different labels was also evaluated by comparing it with other state-of-the-art methods: Baseline I and II described in “Methods” section.

**Figure 3:**
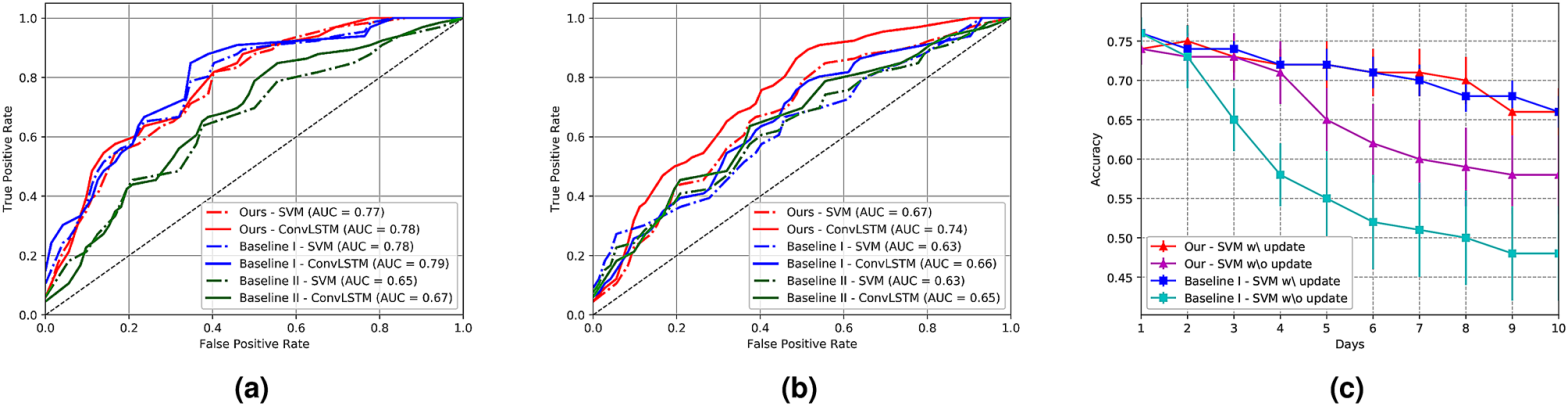
Comparisons of classification results between the proposed system and Baselines I & II methods on the dataset (**a**) $\mathbb {S}_{\zeta }$, (**b**) $\mathbb {S}$, and (**c**) $\mathbb {S}_{\zeta }$ for 10 days.

Figure 3a,b show the evaluation results for the two sets of affective situations. For dataset $\mathbb {S}_{\zeta }$, as shown in Fig. 3a, our system performed comparably to Baseline I. Although it achieved slightly worse results than Baseline I when 0.2 < FP < 0.6, these two methods perform equally well overall on the dataset. These results can be attributed to the fact that the labels provided by our proposed system categorize EEG signals associated with different emotions. Although the predicted labels obtained from our system have different interpretation from the SAM ratings by the Baseline I for rating real-world situations (see Supplementary Materials), the classifiers based on our system achieve similar performance to those based on the SAM ratings. In contrast, the results of the Baseline II method are the worst for all cases; this can be explained by noting that the use of optical flow-based motion components alone has less discriminative power to classify physiological patterns in various situations.

Figure 3b shows the ROC curves for dataset $\mathbb {S}$. Overall, the proposed system performed favorably in classifying emotional states with higher area under the curve (AUC) than any of the baseline methods. Although ConvLSTMs increased the distinctiveness in order to classify EEG signals labeled by Baseline I, the two methods are less discriminative than the proposed method in terms of AUC. This superior performance by our system demonstrates the effectiveness of the proposed system for overcoming intra-subject variability in EEG signals. Figure 3c shows the classification accuracy for 10 days on dataset $\mathbb {S}_{\zeta }$. Note that participants were asked to rate their feelings spontaneously each day if they had encountered any situation where a certain visual content elicited a specific feeling. When updating SAM ratings every day, the two methods (A-Situ and Baseline I) had similar performance (red and blue colored lines). However, the comparative result in preventing the update (magenta and cyan colored lines) shows our parabola-based system has less decrement than the SAM-based system, which suffers from inter-day variability in EEG signals. The classifiers more reliably learn physiological patterns in EEG signals associated with affective states predicted by our model than do those rated by the SAM. Furthermore, these results imply that the proposed system performs robustly in real-world environments with their many different possible situations.

**Figure 4:**
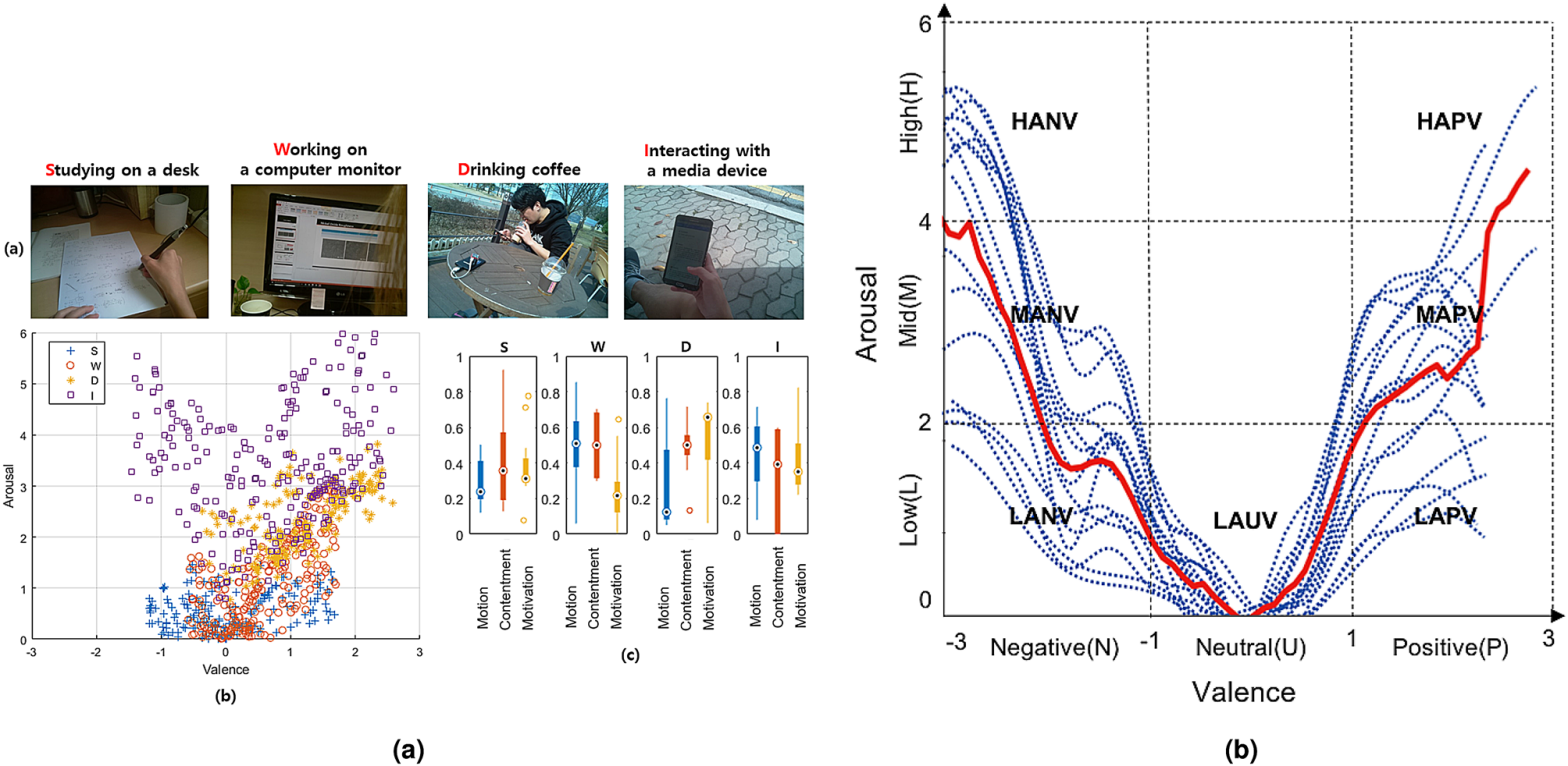
(**a**) The four most frequent affective situations. Example images, accumulated emotion points over valence and arousal points, and the means of the motivation, motion, and contentment components for all participants. (**b**) Affective states subdivided by low (LA), mid (MA), and high (HA) arousal and negative (NV), neutral (UV), and positive (PV) valence ratings, for all participants over different affective states in the dataset $\mathbb {S}$. Dashed lines indicate individual participants, and a solid red line is the mean curve of all participants.

The proposed A-Situ provides affective labels underlying physiological characteristics associated with psychological phenomena. Since the framework outputs a set of affective labels in a spatiotemporal situation, pairs of labels on the affective curve contain emotional traces in response to the affective content of a situation. Figure 4b shows affective curves created by combining the arousal and valence curves in Eqs. (7) and (9). Each curve represents the emotional representation of affective situations in the everyday life of a participant. The parabolic shape of the mean curve covers the VA emotion space, except for some emotions characterized by neutral valence and high-level arousal.

To demonstrate the effectiveness of the model, we show some interesting cases that involve analyzing physiological characteristics. We choose the four most frequent situations: “Working on a computer”, “Studying at a desk”, “Drinking coffee”, and “Interacting with a media device” on the dataset $\mathbb {S}$. Figure 4a shows example images, accumulated valence and arousal labels over valence-arousal dimension, and the three components.

The situation “Studying at a desk” drew affective curves around the valence and arousal values between − 1 and 1 and between 0 and 1.3, respectively; these low scores were due to results from the motion and motivation components rather than the contentment component (see Fig. 4a). This phenomenon indicates that most participants in this situation spent longer sitting stationary than in other situations to keep concentrating while studying. This activity in the situation yielded lower motivation and motion components but a higher contentment component. Negative affect occurs when participants have low motivation and contentment components, leading them to stop studying and leave the situation earlier than usual. Such thwarted goals incur negative feeling such as frustration.

The situation “Working on a computer monitor” drew similar affective curves to “Studying at a desk,” but had larger values for the motion component than the latter. This indicates that interaction with a computer monitor, such as exploring/searching websites, lead to larger motion changes in display than “Studying at a desk”, but smaller than the other activities, with correspondingly higher/lower arousal values.

The situation “Drinking coffee” includes activities whose affective curves were affected by the motivation and contentment components. When participants stayed in their circumstances and drank coffee while interacting with other factors, the two components had high values, resulting in a high valence score. For example, approaching (drinking) a cup of coffee, reading a book, and hanging out with friends led to increased values of the two components, which resulted from the movements of either hand while approaching the coffee cup or other movements during the long sequence of the situation. Since this personalization determines the degree of the valence score, this score was highly variable, with a standard deviation of ± 0.7.

The situation “Interacting with a media device” includes activities where participants interact with several digital media, such as playing PC games, watching YouTube videos, or posting on the social media. This situation had the highest variance in valence and arousal scores, and the contentment and motion components were spread wider than the motivation component. The level of acceptance of frequent motion of objects in media and playing or using them for a long time led to changes in valence and arousal scores. Negative affect such as frustration can occur when participants have low motivation and contentment components, implying loss of interest and leaving the situation earlier than usual when they thwart their own goals, such as through an unexpected loss in a game.

Our empirical study showed that the proposed A-Situ can provide affective labels underlying emotional behaviors based on visual measurement and showed the efficacy of affective curves as a reliable representation for labeling emotions. Since our system is underlain by a particular motivational theory, however, it may not cover all of the complexity or real emotion. For instance, some negative emotions such as anger cannot be measured instantly by our system, since they involve approach to (as opposed to avoidance of) negative stimuli. Some emotions related to high arousal and low movement (i.e., fear and freezing) may not be labeled as the same precision at as the SAM ratings in the valence-arousal dimensional space.

Nevertheless, our system enables people to understand how their emotions change when they feel under the situation, since the proposed labeling system outputs a set of affective labels in a spatiotemporal situation rather than a single universal set of labels. Each participant had their own emotional behaviors to recognize and deal with emotion, and such responses could be represented as continuous pairs of labels on their own affective curve by our system. The pairs of labels on an affective curve contain emotional traces in response to the affective situation, enabling our system to provide a better understanding of affective perception in a situation than existing subjective self-reports do.

## Methods

### Affective situation labeling system

A-Situ defines an affective situation in order to represent and model it as a set of points in the valence-arousal emotion space.

#### Definition 1

*Affective situation* An affective situation $\mathscr {S}^i_t$ is a specific arrangement of affective entities relevant to emotion elicitation in situation *i* at time $t \in T_i$

1 $$\begin{aligned} \mathscr {S}^i_t =(\mathscr {M}^i_t, \mathscr {E}^i_t, T_i), \end{aligned}$$

where $\mathscr {M}^i_t$ is an egocentric image sequence, $\mathscr {E}^i_t$ is an accelerometer sequence, $T_i$ is the length of situation *i*.

**Figure 5:**
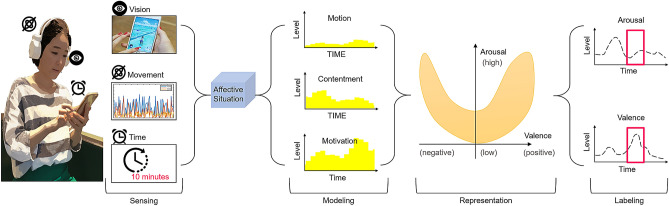
Overview of the proposed A-Situ system. For every timestamp, our system recognizes the expected feeling based on a person’s behaviors in a situation. A set of expected feelings in an affective situation is represented by a curve called affective curve.

Figure 5 shows the entire framework of A-Situ. The system provides affective labeling from an affective situation in real-world scenarios. To quantify the feeling evoked in a situation, A-Situ focuses on learning and representing an affective situation. At each time *t*, our system takes an egocentric image $\mathscr {M}^i_t$ and uses auxiliary accelerometer data $\mathscr {E}^i_t$ sequences as inputs, outputting a set of two emotional points $\mathscr {L}^i_t$ over valence-arousal space. The learned points are represented as a polynomial curve called affective curve.

2 $$\begin{aligned} \hat{\mathscr {L}}_{1:t}={\mathop {{{\,\text{arg\,max}\,}}}_{(\mathscr {V},\mathscr {A}) \in \mathscr {L}}}\,p(\mathscr {L}_{1:t}|\mathscr {S}_{1:t}) \end{aligned}$$

In a given situation, we can observe several affective expressions. The following factors can be used to model these emotional phenomena in terms of arousal and valence:

- *Motion*: The influence of object motion on human emotional response has revealed that an increase in motion intensity causes an increase in arousal^6^.
- *Motivation*: Some theories regard affective valence to be tightly coupled with motivational direction, such that positive affect is associated with approach motivation and negative affect is associated with avoidance motivation^20,28^.
- *Contentment*: Attitudes toward discrete emotions predict emotional situation selection. For instance, more positive attributes toward “excited” are more likely to express interest in adapting “excited”-evoking stimuli with self-contentment^29^.

Based on the three factors, A-Situ produces valence $\mathscr {V}$ and arousal $\mathscr {A}$ values, imposing spatial constraints on valence-arousal space, based on the following criteria.

- *Comparability*: This ensures that the values of arousal, valence, and the resulting affect curve obtained in different situations for similar types of emotional behavior are comparable. This criterion naturally imposes normalization and scaling requirements when computing time curves.
- *Compatibility*: This ensures that the shape of the affect curve reflects the situation at a particular given time in the valence-arousal emotion space. When the situation ends, the appearance of the curve becomes a roughly parabolic contour of the 2D emotion space.
- *Smoothness*: This describes the degree of emotional retention of preceding frames in the current frame. It ensures that the affective ratio of the content related to eliciting human emotions does not change abruptly between consecutive frames of a situation.

The proposed system uses general functions $\mathscr {A}(\mathscr {S})$ and $\mathscr {V}(\mathscr {S})$ for arousal and valence in an affective situation $\mathscr {S}$. The two functions have the appropriate form of functions to integrate the three components: motion, motivation, and contentment components as given above.

#### Motion component of emotional responses

To calculate the motion component $m(\mathscr {S}^i_t)$, A-Situ estimates the motion of objects in situation *i* at time *t*. The system first uses optical flow estimation to characterize and quantify the motion of affective objects between adjacent frames; then, the average magnitude of all estimated motion vectors formulates motion activity

3 $$\begin{aligned} \bar{m}(\mathscr {S}^i_t)=\frac{1}{B|\vec {v}_{max}|}\left( \sum ^B_{k=1}{|\vec {v_k}(t)|}\right) , \end{aligned}$$

where $\vec {v_k}(t)$ is the motion vector *k* and *B* is the number of motion vectors at time *t*. To suppress motion artifacts, we used accelerometer data $\mathscr {E}^i_t$ in the motion component $m(\mathscr {S}^i_t)$.

4 $$\begin{aligned} m(\mathscr {S}^i_t) = (1 - G( \mathscr {E}^i_t ))\cdot \bar{m}(t), \end{aligned}$$

where $G(\cdot )$ is the Gaussian smoothed results normalized between 0 and 1. Note that $1 - G( \mathscr {E}^i_t )$ implies that an increase in motion artifacts causes a decrease in arousal, because motion artifacts are not actual factors of motion activities.

#### Motivation component of emotional approach-withdrawal behaviors

**Figure 6:**
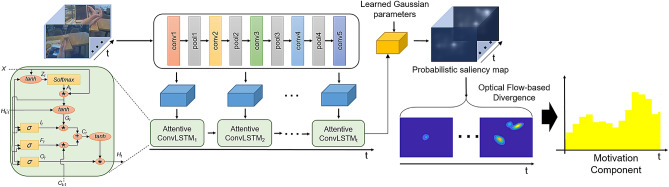
Overview of the motivation component. From an image at each video time *t*, visual attention is detected by the saliency maps. Under the area covered by the binary saliency map, the emotional approach-withdrawal behaviors associated with the attentive object are calculated using optical flow around the object.

The motivation component is derived in two stages (see Fig. 6). It aims to compute emotional saliency within visually attentive areas. We first detect the participant’s intention regarding visual attention. Predicting the location of visual attention maintained at a certain fixation point can be done with saliency prediction or detection. To obtain the most salient region in an image frame, we used the saliency-attentive (SA) model, as in^30^, in which human eye fixations during a scene were predicted by building a convolutional long short-term memory (ConvLSTM) with a set of features computed by dilated convolutional networks (DCN) and multiple learned gaze priors as a salient object detector.

In an affective situation, ConvLSTMs take visual features extracted from images and refine them in the prior learned module. More specifically, they compute an attention map by convolving the previous hidden state and the input, producing the output as a normalized saliency spatial map through the softmax operator in the output layer. Given the final saliency map, which is a probability map with values within [0, 1], we generate a binary saliency map with a threshold $t_h$. Then, the white area in the binary saliency map becomes the prime fixation area to which the participant applies visual attention. Within the area of saliency prediction, we compute emotional saliency after the second stages. As the second stage, we learn the emotional approach-withdrawal behaviors associated with a saliency object in the prime fixation area detected by the first stage. More specifically, we compute divergence and rotation using optical flow around the attentive object at each video frame *t*. An approach to a single object can be identified by zooming in on the object, and this has the same effect on the divergence of flow vectors surrounding the center point of the object^31^. Inversely, avoidance of a single object associated with withdrawal behaviors can be estimated by the convergence, which is tantamount to zooming out from the object.

**Figure 7:**
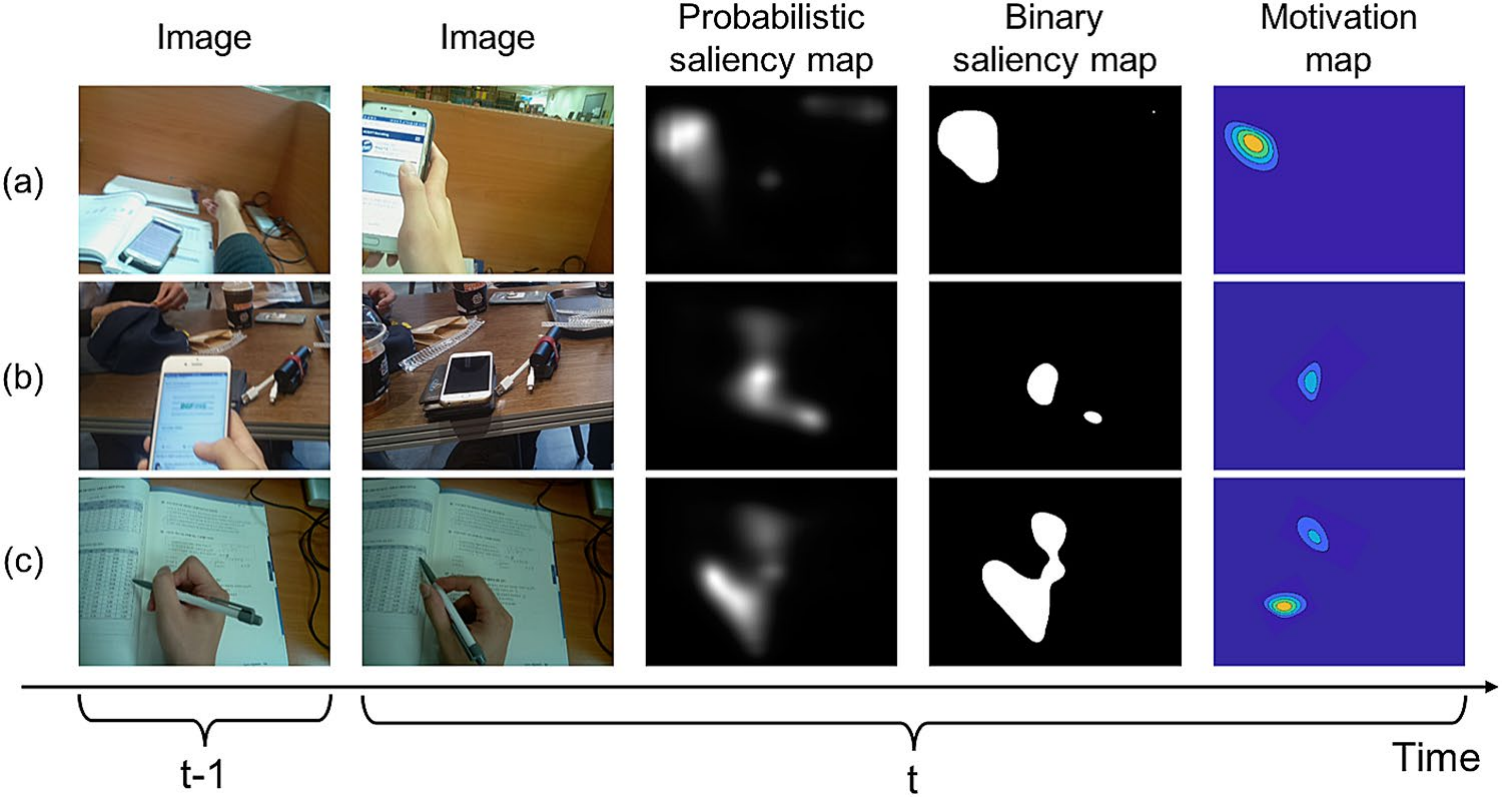
Motivation component examples. (**a**) Raising a hand while interacting with a mobile phone has only positive effects on the value for the component, while (**b**) laying down has the effect of decreasing values in the situation, and (**c**) moving a hand slightly and rotating a pen has a complex motivational effect on the component.

To compute the divergence, we first compute the flow using multi-scale block-based matching between adjacent frames. Then, the flow is standardized as six primitive optical flow patterns^31^: (1) rotation around a vertical axis; (2) rotation around a horizontal axis; (3) approach toward an object; (4) rotation around the optical axis of the image plane; and (5) and (6) complex hyperbolic flows. Given the motion vector field, the velocity of the motion vector field is approximately characterized by six parameters: $u_1, u_2, d_1, d_2, h_1, h_2$. The parameter $u_1$ is associated with right and left rotations, $u_2$ is associated with heading up and down, $d_1$ is associated with approaching the object, and the last three parameters indicate combined motion. Using the six parameters, we compute the motivation component $o(\mathscr {S}^i_t)$ at time *t* as follows:

5 $$\begin{aligned} o(\mathscr {S}^i_t) = \text {exp}\left( \sum _{x=0}^X\sum _{y=0}^Y({d_1}_{(x,y)} / ( {d_2}_{(x,y)} + {h_1}_{(x,y)} + {h_2}_{(x,y)}))\right) , \end{aligned}$$

where *X* and *Y* denote the width and height of the optical flow field of the attentive object. The motivation component $o(\mathscr {S}^i_t)$ in Eq. (5) increases when approaching an object; otherwise, it remains near zero. For instance, raising a hand while interacting with a mobile phone has only positive effects on the values for the component, but laying down decreases values in the situation (Fig. 7). Further detailed information about the motivation component computation is described in Supplementary Materials.

#### Contentment component of an affective situation

We used time-varying situation lengths to reveal a connection between a user’s emotion and his/her intent to adapt to a situation, reflecting self-contentment. We model the emotional contentment of the situation by deriving the function $l(\mathscr {S}^i_t)$ at time *t* as follows:

6 $$\begin{aligned} l(\mathscr {S}^i_t) = \lambda _1 \log ( \delta (t) - \lambda _2 ) + \lambda _3, \end{aligned}$$

where $\lambda _1, \lambda _2$, and $\lambda _3$ determine the shape of the function $l(\mathscr {S}^i_t)$. $\delta (t)$ is the length of situation *i*. The function has logistic growth until the maximum length $T_i$ while staying in situation *i*.

#### Arousal and valence model

To model arousal, the function $A(\mathscr {S}^i_t)$ uses the weighted averages to integrate the contribution of the motion $m(\mathscr {S}^i_t)$ and contentment $l(\mathscr {S}^i_t)$ along with an image sequence in an affective situation at time *t*. The function is convolved with a sufficiently long smoothing window to merge neighboring local maxima of the components through a moving average filter; the result is normalized to a range of 0 to 1.

7 $$\begin{aligned} A(\mathscr {S}^i_t) = \alpha _1\frac{m(\mathscr {S}^i_t)}{m_{max}} + \alpha _2 \frac{l(\mathscr {S}^i_t)}{l_{max}}, \end{aligned}$$

where $\alpha _w$ are the coefficients for weighting the two functions with $\sum _{w=1}^2 \alpha _w = 1$.

The compatibility criterion requires that the affect curve generated by combining the arousal and valence time should cover an area in the valence-arousal coordinate system that has a parabolic shape resembling the 2D emotion space. Clearly, this criterion requires the values of arousal and absolute values of valence to be related; thus, in general, the range of arousal values determines the range of absolute valence values^14^. We, therefore, start the development of the valence model by defining the function $r(\mathscr {S}^i_t)$ that captures this value range dependence considering the value of arousal $A(\mathscr {S}^i_t)$ at the current time *t*

8 $$\begin{aligned} r(\mathscr {S}^i_t)= & {} sign(l(\mathscr {S}^i_t))A(\mathscr {S}^i_t), \end{aligned}$$

9 $$\begin{aligned} V(\mathscr {S}^i_t)= & {} \nu _1 \frac{r(\mathscr {S}^i_t)}{r_{max}} + \nu _2 \frac{o(\mathscr {S}^i_t)}{o_{max}}, \end{aligned}$$

where $r(\mathscr {S}^i_t)$ implies that the negativity of the expected feeling mainly is determined by the amount of emotional contentment in a situation. If a subject wants to avoid the situation, $l(\mathscr {S}^i_t)$ would become a small value and the expected feeling would tend to be negative. Based on this function $\mathscr {A}(\mathscr {S}^i_t)$, the valence value $\mathscr {V}(\mathscr {S}^i_t)$ is determined by the motivation component $o(\mathscr {S}^i_t)$. The function $\mathscr {V}(\mathscr {S}^i_t)$ is smoothed with the same moving average filter as the function $\mathscr {A}(\mathscr {S}^i_t)$. Note that $\nu _i$ are the weighted averages of $r(\mathscr {S}^i_t)$ and $\mathscr {V}(\mathscr {S}^i_t)$, respectively.

#### Affective situation representation

We represent affective situations over 2D emotion space from valence and arousal values learned by the above calculation. We used the set of two emotional values to fit a Gaussian process regression (GPR) model, which is a nonparametric kernel-based probabilistic model that uses a linear basis function and the exact fitting method to estimate the parameters of the GPR model. This results in the production of the affective curve as a representation of an emotional trace along a situation, as perceived by a human.

### EEG preprocessing and setup for classification

As a preprocessing step, high-pass filtered with a 2-Hz cutoff frequency using the EEGlab toolbox and the same blind source separation technique for removing eye artifacts were applied. A constrained independent component analysis (cICA) algorithm was applied to refine the signal removing motion artifacts^32^. The cICA algorithm is an extension of ICA and has been applicable in cases in which prior knowledge of the underlying sources is available^33^.

EEG signals are vulnerable to motion artifacts^34^. Rather than separating and removing motion artifacts in EEG signals occurred by body movement^35,36^, we developed a strategy to get better-quality EEG signals by abandoning signals highly correlated with motion artifacts. To execute this strategy, we subdivided EEG signals into two groups separated by the accelerometer data $\mathscr {E}^i_t$ ranged from 0 to 1. From each of the two groups, we extract the following EEG features: (1) mean power, (2) maximum amplitude, (3) standard deviation of the amplitude, (4) kurtosis of the amplitude, and (5) skewness of the amplitude. These features are metrics to describe the key characteristics of clean EEG^37^. After representing the features in two-dimensional space using principal component analysis (PCA), we compute the Bhattacharyya distance between the two groups over the two-dimensional space. The optical $G(\cdot )$ is determined as a differentiator between the clean EEG and the contaminated EEG, based on the maximum distance between the two groups.

Recent studies on extracting EEG-based features in emotion recognition have categorized these features into three domains: time, timefrequency, and frequency^8^. Among these, frequency domain features have been the most popular, assuming that the signal remains stationary for the duration of a trial. Hence, we used frequency domain features introduced in^38^: higher-order spectra (HOS) and power spectral density (PSD) features in different frequency bands. HOS features have been used to analyze human emotion as a spectral representation of higher-order moments or cumulants of a signal^38^. Specifically, we used the mean of bicoherence in four frequency bandstheta (4–7 Hz), alpha (8–13 Hz), beta (14–29 Hz), and gamma (30–45 Hz) to study the efficacy of affective labels to categorize EEG signals. Bicoherence is the normalized bispectrum of a signal *x*(*t*). Signals are divided into 1-s non-overlapping segments. Within each segment, data are Hanning windowed and Fourier transformed. Then, the bispectrum $B(\omega _1, \omega _2)$ is mathematically defined as

10 $$\begin{aligned} B(\omega _1, \omega _2) = X(\omega _1)X(\omega _2)X^*(\omega _1 + \omega _2), \end{aligned}$$

where $X(\omega )$ is the Fourier transform of the signal *x*(*t*) and $X^*(\omega )$ is its complex conjugate. Note that the bispectrum preserves phase information of the different components of the signal *x*(*t*). Two frequency components $X(\omega _1)$ and $X(\omega _2)$ are phase coupled when there exists a third component at a frequency of $\omega _1 + \omega _2$. The bicoherence $b_c(\omega _1, \omega _2)$ is defined as

11 $$\begin{aligned} b_c(\omega _1, \omega _2) = \frac{|B(\omega _1, \omega _2)|}{\sqrt{P(\omega _1)P(\omega _2)P(\omega _1+\omega _2)}}, \end{aligned}$$

where $P(\omega _i)$ is the power spectrum at $\omega _i$. It quantifies the extent of phase coupling between two frequency components. The resulting frequency resolution is 1 Hz on at both the $\omega _1$ and $\omega _2$ axis. The mean magnitude of $b_c(\omega _1, \omega _2)$ in the four frequency bands is computed as

12 $$\begin{aligned} b_c^{avg}(q_1,q_2) = \frac{1}{L}\sum _{q_1}\sum _{q_2}b_c(q_1, q_2), \end{aligned}$$

where $q_1$ and $q_2$ are frequency bands and $L_{(q_1,q_2)}$ is the number of frequency components in $q_1$ and $q_2$. Power features of the PSD are estimated using Welchs method^38^ and divided into the four frequency bands. The $b_c^{avg}$ and the mean power of the four frequency bands are used to analyze the correlates of the affective labels with EEG signals.

#### Evaluated methods

The efficiency of affective labels provided by our system to discriminate different states in EEG-based emotion recognition was evaluated by subject-dependent classification performance using HOS and PSD features through a fivefold cross-validation scheme for all participants. As shown in Fig. 4b, we subdivided continuous affective labels over the valence-arousal space into discrete emotional states: low (0 to 2), mid (2 to 4), and high (4 to 6) for arousal and negative (− 3 to − 1), neutral (− 1 to 1), and high (1 to 3) for valence. We should note that the results of the ANOVA tests for the bicoherence magnitudes and the PSD in the four frequency bands of the affective states were low *p*-values (lower than 0.05), except the beta frequency band (*p* = 0.0679). The *p*-values resulted from the bicoherence magnitudes in all frequency bands, and PSD in the theta, alpha and gamma frequencies indicated that the three frequency bands appear to be significantly different from emotional states. These results imply that PSD and bicoherence can be used effectively as physiological features to classify emotions.

For the classification process, we choose two classifiers: a support vector machine (SVM) and a ConvLSTM, both of which have been used widely in emotion recognition^8^. 1 min before and after events was used for the evaluation. For SVMs, we extract the PSD and bicoherence features $b_c(\omega _1, \omega _2)$ in the four frequency bands, use mutual information with the maximal relevance criteria for feature selection, and take the top two features as input for classification. For ConvLSTMs, the PSD features in the four frequency bands are fed into ConvLSTMs, as in^17^ to classify the affective states. ConvLSTMs are configured with 3-layer networks with 64 hidden states and the input-to-state and state-to-state kernel sizes of $5 \times 5$. We used learning batches of 16 sequences. Back-propagation through time was performed for ten timesteps. The momentum and weight decay were set to 0.9 and 0.0005, respectively. The learning rate starts at 0.01 and is divided by 10 after every 20,000 iterations. We also performed early-stopping on the validation set. The above configuration was chosen as the best configuration, which yielded the minimum loss in the training set.

To compare classification results, the following models are trained by the two classifiers and evaluated on Affective Situation Dataset $\mathbb {S}$.

- *Baseline I* The model is trained on the dataset $\mathbb {S}_{\zeta }$; which labels in affective situations were rated by the SAM. To compare the performance with the other two methods, the model is evaluated on the datasets of both $\mathbb {S}_{\zeta }$ and $\mathbb {S}$.
- *Baseline II* For the algorithm, like^6^, we replace shot lengths, which is a number of consecutive frames with $T_i$ for situation *i*. The sound energy and pitch-average components are excluded from computation of affective labels, since dataset $\mathbb {S}$ does not include any sound. The model is trained and evaluated on both $\mathbb {S}_{\zeta }$ and $\mathbb {S}$. Since the model only rates arousal labels, evaluation is carried out to classify affective states associated with arousal: low-arousal (LA), mid-arousal (MA), and high-arousal (HA) states.
- *Our proposed model* Our proposed model is trained, and evaluated on both $\mathbb {S}_{\zeta }$ and $\mathbb {S}$, for which labels were computed by A-Situ.

## Conclusion

Here, we presented a computational framework called A-Situ that provides affective labels for real-life situations, defining the term “affective situation” as a specific arrangement of affective entities people encounter, interact with, and which elicit some emotional response in the people. Our system showed efficacy at capturing EEG-based physiological characteristics and understanding psychological behaviors as measured by our proposed wearable device, based on real-world experiments. Modeling affective situations allows us to better understand the contents of human interactions, and representing these situations can determine the level of an interactants expected feelings based on the interaction. Therefore, our framework helps to bridge the semantic gap between cognitive and affective perception in real-world situations.

## Supplementary information

Supplementary Information
