# Supplementary material for: A-Situ: a computational framework for affective labeling from psychological behaviors in real-life situations
Source: Sci Rep. 2020 Sep 28;10:15916. doi: 10.1038/s41598-020-72829-3 (PMC7522975; doi:10.1038/s41598-020-72829-3)
Supplement: Supplementary file 1 — Supplementary Information [file 41598_2020_72829_MOESM1_ESM.pdf]

Supplementary

Byung Hyung Kim[1], Sungho Jo[1], and Sunghee Choi[1,*]

[1]School of Computing, KAIST, Republic of Korea
[*]Corresponding author. E-mail: sunghee@kaist.ac.kr

ABSTRACT

This paper provides supplementary materials for our work titled “A-Situ: a computational framework for affective labeling from psychological behaviors in real-life situations”. It includes more detailed information on Affective Situation Dataset, Affective Situation Labeling System, and EEG analysis.

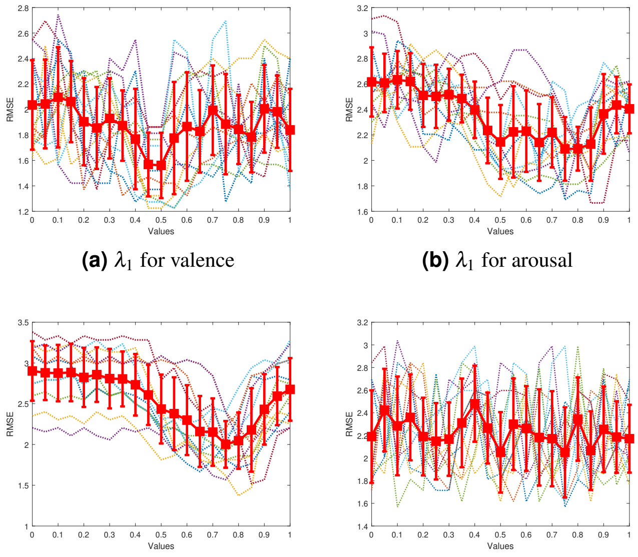

(a) λ_1 for valence **(b)** λ_1 for arousal

(c) α_1 **(d)** ν_1

Figure 1. Accuracy results of valence and arousal ratings by the proposed system with respect to (a) and (b)λ_1, (c)α_1, and (d)ν_1

Affective Situation Dataset

Participant Self-Assessment (SAM-rated Situation Dataset)

Participants were asked to rate their feelings spontaneously each day if they had encountered any situation where a certain visual content elicited a specific feeling. In our work, visual content is gathered by the proposed wearable device–book, coffee cup, media device including cell phone, research paper, or monitor. In addition, every five days, we manually retrieved unrated situations containing the visual content which the participant had chosen previously as emotional stimuli and asked the users to rate their feelings in the situation if they could recall them.

Affective Situation Labeling System

Parameter Settings and Label Comparision

To model affective situations and represent them as affective curves, the parameters introduced in Section 3 for each participant were set through a five-fold cross-validation scheme, except λ_2 and λ_3. These parameters were set to 2 and -1, indicating that the contentment component has a minimum value of $-1/l_{max}$ at the first frame in a situation. m_{max}, l_{max}, r_{max}, and o_{max} are determined by the maximum values during every five days.

Figure 1 shows the results from the five-fold cross-validation in terms of valence and arousal rating distances between the SAM and A-Situ with respect to different parameters α_1, λ_1, and ν_1. Because an affective curve represents affective dynamics in a spatiotemporal situation and consists of multiple pairs of affective labels, it is difficult to directly compare them with a pair of SAM ratings, in which the valence and the arousal ratings are a pair of discrete values for representing an emotion in the same situation. For comparison, we calculate root-mean-squared-errors (RMSEs) of a pair of affective labels scaled 0 to 6 over all participants, as follows:

$$RMSE = \sqrt{\frac{1}{N_s}\sum_{i=1}^{N_s}(\hat{y}_i - y_i)^2}, \quad (1)$$

where N_s is the numbers of situations in $\mathbb{S}_\zeta$, $\hat{y}_i$ is the mean value of pairs of the predicted affective labels ($\mathscr{V}$, $\mathscr{A}$), and y_i is a pair of ground truth labels in situation $i \in \mathbb{S}_\zeta$. Note that 0 represents negative, 3 neutral, and 6 positive valence ratings; and 0 represents neutral, 3 represents low, and 6 represents high arousal ratings.

While α_w and λ_w ($w = 1, 2$) determine the arousal value $\mathscr{A}(\mathscr{S}_t^i)$, the valence value $\mathscr{V}(\mathscr{S}_t^i)$ is determined by the parameters λ_w and ν_w. The parameter λ_1 determines when the contentment component $l(\mathscr{S}_t^i)$ becomes zero; this directly affects the

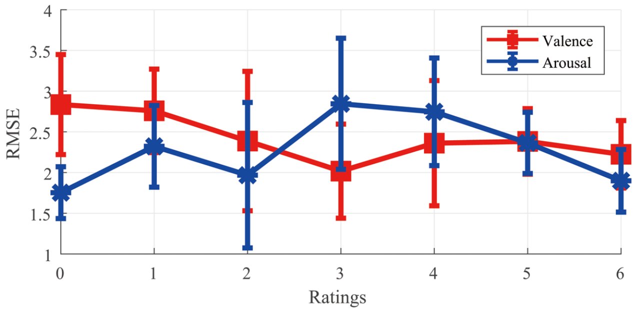

Figure 2. Mean RMSEs of all SAM-rated situations on dataset $\mathbb{S}_\zeta$ between predicted labels and ground truth labels, scaled 0 to 6 over valence (red) and arousal (blue) dimensions. Note that 0 represents negative, 3 neutral, and 6 positive valence ratings, while 0 represents neutral, 3 low, and 6 high arousal ratings.

sign of the valence value $\mathscr{V}(\mathscr{S}_t^i)$ and increment of the arousal value $\mathscr{A}(\mathscr{S}_t^i)$. With a smaller λ_1 value, the contentment component $l(\mathscr{S}_t^i)$ becomes zero and the valence sign $\mathscr{V}(\mathscr{S}_t^i)$ becomes positive at an earlier t. As shown in Figure 1a, the highest performance on valence ratings was 0.5, and two other points, 0.25 and 0.75, were the next highest parameters for valence ratings. Conversely, the distances between arousal ratings are minimized after 0.5 for most participants, as shown in Figure 1b. The parameter α_1 and its counterpart $\alpha_2(=1-\alpha_1)$ determine the level of arousal in terms of the motion and contentment components. The results reveal that the proposed system has the best performance for arousal ratings at between 0.7 and 0.8 for most participants (see Figure 1c) with respect to α_1. Distances beyond this range remain almost the same when α_1 is greater than 0.85 and less than 0.4. The parameter ν_1 and its counterpart $\nu_2(=1-\nu_1)$ determine the level of valence. With a large value of ν_1, the resulting valence may not properly represent the emotion associated with approach-withdrawal behaviors. As shown in Figure 1d, the performance of our proposed system fluctuates significantly when the parameter ν_1 is varied. We set α_1 and ν_1 to be 0.75 and 0.5 for all participants and λ_1 was chosen from 0.25, 0.5, and 0.75 to yield the minimum distance for each individual in each of the following experiments.

Figure 2 shows the mean RMSEs of all SAM-rated situations on dataset $\mathbb{S}_\zeta$ between labels predicted by the proposed system and ground truth labels as rated by the SAM. Note that 0 represents negative, 3 neutral, and 6 positive valence ratings, while 0 represents neutral, 3 low, and 6 high arousal ratings. The mean accuracies for valence and arousal ratings between the two labels were respectively 2.42 ($\pm$0.59) and 2.27 ($\pm$0.7), equivalent to 59.7% and 62.2% in terms of normalized RMSE. In both cases, neutral ratings had smallest errors; while higher ratings on arousal had more similarity, negative ratings on valence had larger errors with larger standard deviations. We should affirm that A-Situ does not aim to evaluate affective labels at the same precision level as the SAM ratings; instead, the primary purpose of the proposed system is to provide reliable emotion labels associated with physiological characteristics derived from psychological behaviors.

Motivation Component of Emotional Approach-Withdrawal Behaviors

To compute the divergence, we first compute the flow using multi-scale block-based matching between adjacent frames. Then, the flow is standardized as six primitive optical flow patterns[1]: 1) rotation around a vertical axis; 2) rotation around a horizontal axis; 3) approach toward an object; 4) rotation around the optical axis of the image plane; and 5) and 6) complex hyperbolic flows. Given the motion vector field, the velocity e_p at the pixel $p(x,y)$ can be represented as

$$e_p = e_{p_0} + \bar{\chi}(p - p_0), \tag{2}$$

where $e_{p_0} = (u_1, u_2)^T$ is the velocity at pixel p_0 and $\bar{\chi}$ is the matrix defined as

$$\bar{\chi} = \begin{bmatrix} \chi_1 & \chi_3 \\ \chi_2 & \chi_4 \end{bmatrix}. \tag{3}$$

Then $\bar{\chi}$ can be decomposed to

$$\bar{\chi} = \frac{1}{2}(d_1 D_1 + d_2 D_2 + h_1 H_1 + h_2 H_2), \tag{4}$$

where the four matrices are defined as follows:

$$D_1 = \begin{bmatrix} 1 & 0 \\ 0 & 1 \end{bmatrix}, D_2 = \begin{bmatrix} 0 & -1 \\ 1 & 0 \end{bmatrix}, \qquad H_1 = \begin{bmatrix} 1 & 0 \\ 0 & -1 \end{bmatrix}, H_2 = \begin{bmatrix} 0 & 1 \\ 1 & 0 \end{bmatrix}, \tag{5}$$

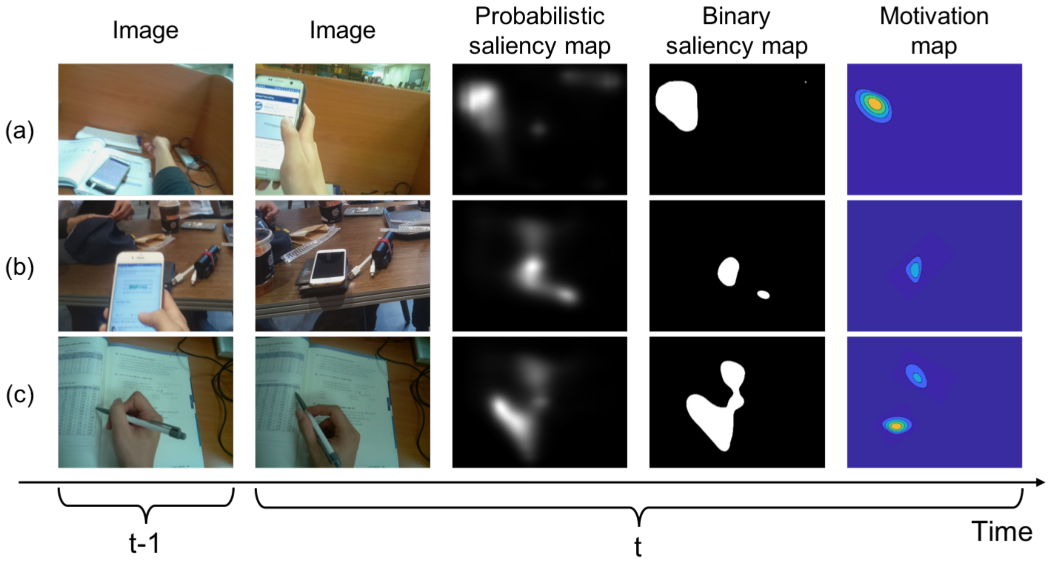

Figure 3. Motivation component examples. (a) Raising a hand while interacting with a mobile phone has only positive effects on the value for the component, while (b) laying down has the effect of decreasing values in the situation, and (c) moving a hand slightly and rotating a pen has a complex motivational effect on the component.

where $d_1 = \chi_1 + \chi_4$ and $d_2 = \chi_2 + \chi_3$ refer to divergent and rotating optical flows, respectively. $h_1 = \chi_1 - \chi_4$ and $h_2 = \chi_2 + \chi_3$ refer to different types of hyperbolic optical flows. The velocity of the motion vector field can be approximately characterized by six parameters: $u_1, u_2, d_1, d_2, h_1, h_2$. Given an optical flow field of the attentive object, we estimate the parameter vector using (4) and the least squared error method. The parameter u_1 is associated with right and left rotations, u_2 is associated with heading up and down, d_1 is associated with approaching the object, and the last three parameters indicate combined motion.

Using the six parameters, we compute the motivation component $o(\mathscr{S}_t^i)$ at time t as follows:

$$o(\mathscr{S}_t^i) = \exp(\sum_{x=0}^{X}\sum_{y=0}^{Y}(d_{1(x,y)}/(d_{2(x,y)} + h_{1(x,y)} + h_{2(x,y)}))), \tag{6}$$

where X and Y denote the width and height of the optical flow field of the attentive object. The motivation component $o(\mathscr{S}_t^i)$ in (6) increases when approaching an object; otherwise, it remains near zero. For instance, as shown in Figure 3a, raising a hand while interacting with a mobile phone has only positive effects on the values for the component, but laying down decreases values in the situation. Figure 3b shows minimal values reflecting non-emotional behaviors.

Wearable Device Configuration

We designed a simple, easily wearable device such that users could act freely in everyday situations while the device simultaneously, correctly collects their emotions. Since human affect is sophisticated and subtle, it is vulnerable to personal, social, and contextual attributes. The noticeability and visibility of wearable devices could elicit unnecessary and irrelevant emotions, while recording of human affect should be unobtrusive when measured in the natural environment. To design an unobtrusive device, we imitated the design of existing easy-to-use wireless headsets. We note that the term “unobtrusive device” means that it is not easily noticed or does not draw attention to itself; it does not imply that our device aims to be small or concealable. This easy-to-use device provides comfort and performance to users during long-term activities.

Our device consists of multimodal sensors to capture various affects surrounding daily life as follows:

- Frontal Camera for Collecting Visual Content: Visual information has been widely used to detect situations faced by an experimental participant. Analysis of scenes and activities in camera images has provided understanding of this contextual information. Hence, in our system, a small frontal viewing camera with a 30 fps sampling rate was used to record the images.
- Small Physiological Sensor to Capture Human Affect: Patterns of physiological changes have been increasingly analyzed in the context of affect recognition. To evaluate the reliability of the affective labels predicted by our system, we analyzed

the distinctiveness of physiological signals as categorized by the predicted labels. We used a two-channel EEG sensor for the left and right hemispheres, with sampling rates of 250 Hz using OpenBCI, a tool that has been applied successfully in several works[2,3].

Analysis of Physiological Characteristics

We have shown the efficacy of affective curves as reliable emotion labels. However, this finding is limited to the clarification of affective labels reflecting physiological characteristics; that is, it only shows the distinctiveness of EEG signals associated with the labels, which were produced based on psychological measurements. Therefore, we investigated the statistical relationship between EEG spectral power in the four frequency bands from two electrodes (F3, F4) and the affective labels for bridging the gap between psychological measurements and physiological evidence.

Table 1. Mean Spearman's correlation coefficients of valence and arousal ratings with EEG spectral power in the four frequency bands (theta, alpha, beta, and gamma) for all participants.

	Valence		Arousal	
Frequency	F3	F4	F3	F4
Theta	0.31(±0.04)	0.22(±0.11)	0.12(±0.09)	0.17(±0.08)
Alpha	**0.37**(±0.11)	**0.26**(±0.12)	**0.22**(±0.15)	**0.26**(±0.05)
Beta	0.04(±0.06)	0.17(±0.05)	0.02(±0.14)	0.11(±0.05)
Gamma	0.12(±0.08)	0.21(±0.12)	0.17(±0.08)	0.23(±0.09)

Table 1 indicates that the alpha frequency components have higher correlations to both ratings than the other frequencies. Since our dataset contains participants' hand movements, the motor cortex activation related to the movements can be correlated with either valence or arousal ratings, because Mu rhythms (8–11 Hz) activated by movements in the motor cortex area have strong associations with the alpha frequency components.

Table 2. Mean Pearson's correlation coefficients of the three components (motion, motivation, and contentment) with EEG spectral power in the four frequency bands (theta, alpha, beta, and gamma) for all participants.

	Motion		Motivation		Contentment	
Frequency	F3	F4	F3	F4	F3	F4
Theta	0.14	0.08	0.19	0.13	0.05	0.07
Alpha	**-0.08**	**0.01**	0.21	0.18	**0.16**	**0.13**
Beta	0.05	-0.07	-0.08	-0.05	0.03	0.09
Gamma	0.27	0.21	-0.12	0.03	0.11	0.05

However, this does not imply psychological measurements in hand movements are uncorrelated with physiological changes. To determine if the alpha frequency band is contaminated by hand movements or correlated with psychological measurements in action, we computed correlation coefficients between the power of the four frequency bands and the three components. Table 2 shows the correlation between physiological brain activity and the affective states, for each component. As we described in Section 3, the motion and motivation components may potentially be influenced by the movements; however, only the motivation component reflects a subject's approach behaviors in psychology and quantifies them in valence ratings. Together with the coefficient values in the alpha frequency band associated with the motion and motivation components, the association between physiological changes in the alpha frequency band and psychological measurements from the movements cannot be a result of motor cortex activation, since Mu rhythms (8–11 Hz) are related to the activation and were only positively associated with the motivation component, and not with the motion component.

The characteristics of the brain signals under different affective labels were investigated and analyzed by the above correlation. EEG-based statistical analysis revealed that physiological responses correlate to continuous affective labels.

References

1. Li, L., Chen, Y., Hu, W., Li, W. & Zhang, X. Recognition of semantic basketball events based on optical flow patterns. In *International Symposium on Visual Computing*, 480–488 (Springer, 2009).

2. Kaongoen, N., Yu, M. & Jo, S. Two-factor authentication system using p300 response to a sequence of human photographs. *IEEE Transactions on Syst. Man, Cybern. Syst.* **50** (2020).

3. Kaongoen, N. & Jo, S. A novel hybrid auditory bci paradigm combining assr and p300. *J. Neurosci. Methods* **279**, 44–51 (2017).
